# The functional limits of the aneurysmal aortic root. A unique pressure testing apparatus

**DOI:** 10.1186/s13019-020-01288-8

**Published:** 2020-09-17

**Authors:** Timothy Luke Surman, John Matthew Abrahams, Dermot O’Rourke, Karen Jane Reynolds, James Edwards, Michael George Worthington, John Beltrame

**Affiliations:** 1 D’Arcy Sutherland Cardiothoracic Surgical Unit, Royal Adelaide Hospital, Adelaide, South Australia Australia; 2 Medical Device Research Institute, College of Science & Engineering, Flinders University, Adelaide, South Australia; 3 Cardiology Department, Queen Elizabeth Hospital, Adelaide, South Australia

**Keywords:** Ascending aorta aneurysms, Ascending aortic dissection, Aortic root, Thoracic aorta, Inflation testing

## Abstract

**Background:**

The aortic root has unique embryological development and is a highly sophisticated and complex structure. In studies that report on the biomechanical characteristics of the thoracic aorta, distinction between the aortic root and ascending aorta regions is nonexistent. Our objective is to determine the maximal pressures at which dissection occurs or tissue failure occurs in the aortic root compared to that of the ascending aorta in the presence of aortic aneurysms. This may help guide preoperative monitoring, diagnosis and the decision for operative intervention for aortic root aneurysms in the normal and susceptible populations.

**Methods:**

We developed a simple aortic root and ascending aorta pressure testing unit in series. Ten fresh porcine hearts were obtained from the local abattoir (*n* = 5 aortic root and *n* = 5 ascending aorta for comparison). Using a saline filled needle and syringe, artificial fluid-filled aneurysms were created between the intima and medial layers of the aortic root. The aorta lumen was then progressively filled with saline solution. Pressure measurement was taken at time of loss of tissue integrity, obvious tissue dissection or aneurysm rupture, and the tissue structure was then visually examined.

**Results:**

In the aortic root, mean maximal pressure (mmHg) at tissue failure was 208 mmHg. Macroscopic examination revealed luminal tears around the coronary ostia in 2/5 specimens, and in all specimens, there was propagation of the dissection in the aortic root in a circumferential direction. In all ascending aorta specimens, the maximal aortic pressures exceeded 300 mmHg without tissue failure or dissection, and eventual apparatus failure.

**Conclusion:**

Our results indicate that the aneurysmal aortic root tissues are at greater risk of rupture and dissection propagation at lower aortic pressure. With further analysis, this could guide clinical and surgical management.

## Background

Ascending aortic dissection is the most common catastrophe of the aorta; it is two to three times more common than rupture of the abdominal aorta [1]. Mortality rate of untreated acute dissection involving the ascending aorta is about 1–2% per hour during the first 48 h [2] The first documented case was King George II in 1760 [2]. Constant exposure to high pulsatile pressure and shear stress leads to a weakening of the aortic wall in susceptible patients resulting in an intimal tear [3] Most of these tears take place in the ascending aorta, usually in the right lateral wall where the greatest shear force on the aorta occurs [4].

Aneurysms of the aortic root arise relatively deep within the heart and because of frequently associated complications, such as aortic insufficiency, present a more complicated problem than the more distal aneurysms of the ascending aorta [5]. The aortic root has unique embryological development and is a highly sophisticated and complex structure. Its optimal structure ensures dynamic behavior in flow characteristics, coronary perfusion and left ventricular function. In studies that report on the biomechanical characteristics of the thoracic aorta, distinction between the aortic root and ascending aorta regions is nonexistent. Aortic root replacement is associated with high mortality and morbidity and is therefore frequently avoided in cases of acute aortic dissection for fear of increased surgical risk. Approximation of the aortic wall layers within the dissected sinuses of Valsalva with a biological glue and subsequent supracoronary aortic replacement offers a simple and efficient method of preserving the native valve and abolishing the aortic insufficiency when it is caused by the distortion of root anatomy. However, non-curative root repair can result in late development of several pathologies, which, especially after use of glue, necessitate challenging redo surgeries [6].

The initial decision regarding the management of the aortic root in type A aortic dissection (TAAD) is whether to repair or replace the dissected sinus segments [7]. The standard indications for aortic root replacement (ARR) in the setting TAAD are extensive tissue destruction, the presence of a concomitant aortic root aneurysm ≥4.5 cm, or a known connective tissue disorder. The most common pathology observed is a primary intimal tear located in the ascending aorta with extension of the dissection flap into the noncoronary cusp, and relative preservation of the left and right coronary sinuses. Rarely are the aortic valve cusps or annulus impacted by the dissection process [7].

A meta-analysis of aortic valve-preserving surgery in acute type A aortic dissection containing 2402 patients from 19 observational studies revealed that, in 95% of the patients, the surgery consisted of conservative root management and supracoronary aortic replacement, while only 5% underwent a curative root repair by valve-sparing root replacement (VSRR) (reimplantation or remodeling). In a large aortic dissection repair centre, 10% of the patients with aortic root dissection, a non-curative root repair using tissue glue was performed at the surgeon’s discretion [6].

Coady et al. studied 370 patients with thoracic aneurysms (201 ascending aortic aneurysms), during a mean follow-up of 29.4 months, the incidence of acute dissection or rupture was 8.8% for aneurysms less than 4 cm, 9.5% for aneurysms of 4 to 4.9 cm, 17.8% for 5 to 5.9 cm, and 27.9% for those greater than 6 cm. In this study, the median size of the ascending aortic aneurysm at the time of dissection or rupture was 59 mm. The growth rate ranged from 0.08 cm/yr. for small (4 cm) aneurysms to 0.16 cm/yr. for large (8 cm) aneurysms [8].

The risk of aortic dissection and rupture is often related to the transverse diameter of the aortic sinuses. It is rare with diameters less than 50 mm except in cases of family history of dissection or inpatients with Loyes-Dietz syndrome. Surgery is usually recommended when the diameter of the aortic root reaches 50 mm. Patients with family history of aortic dissection or the diagnosis of Loyes-Dietz syndrome should be operated on when the transverse diameter exceeds 40 mm [8].

Our objective is to determine the maximal pressures at which dissection occurs or tissue failure occurs in the aortic root compared to that of the ascending aorta in the presence of aortic aneurysms. This may help guide preoperative monitoring, diagnosis and the decision for operative intervention for aortic root aneurysms in the normal and susceptible populations.

## Methods

**Fig. 1:**
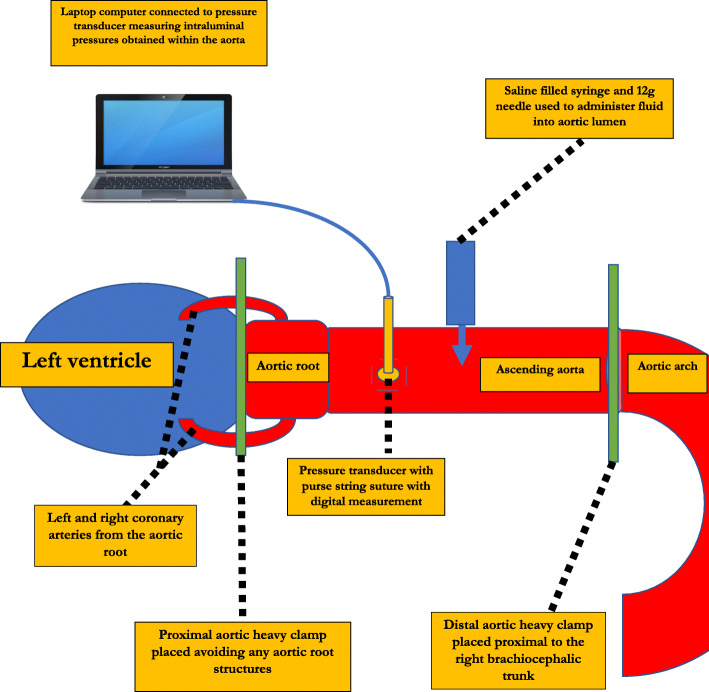
Diagram of the aortic root and ascending aorta pressure apparatus. This diagram is labelled with the main features of the apparatus. Two clamps are placed proximal and distal to isolate the aortic root and ascending aorta. The administration of saline into the lumen of the aorta and the pressure transducer connected to a nearby laptop to measure and record the maximal pressures before aortic or apparatus failure is demonstrated

We developed a simple aortic root and ascending aorta pressure testing unit in series (Fig. 1). This apparatus consisted of an aortic root and ascending aorta porcine specimen, a pressure transducer measuring in mmHg (National instruments Pty Ltd., Austin, TX), two large vessel clamps, and a 50 ml syringe filled with saline solution with a 21-gauge needle.

Porcine hearts (*n* = 5) were obtained fresh from local abattoirs which included the heart and ascending aorta attached to the brachiocephalic trunk on the right side. In addition, porcine hearts (*n* = 5) were obtained for testing on the ascending aorta alone (excluding the aortic root). Animal ethics approval was not required according to local South Australian Health and Medical Research Institute (SAHMRI) and Preclinical, Imaging, and Research Laboratories (PIRL) protocols.

The aorta was dissected proximally to the left ventricle to include the entire aortic root. The dissection then extended distally to the distal ascending aorta. The proximal limits were the left ventricle and distal limits was the brachiocephalic trunk.

**Fig. 2:**
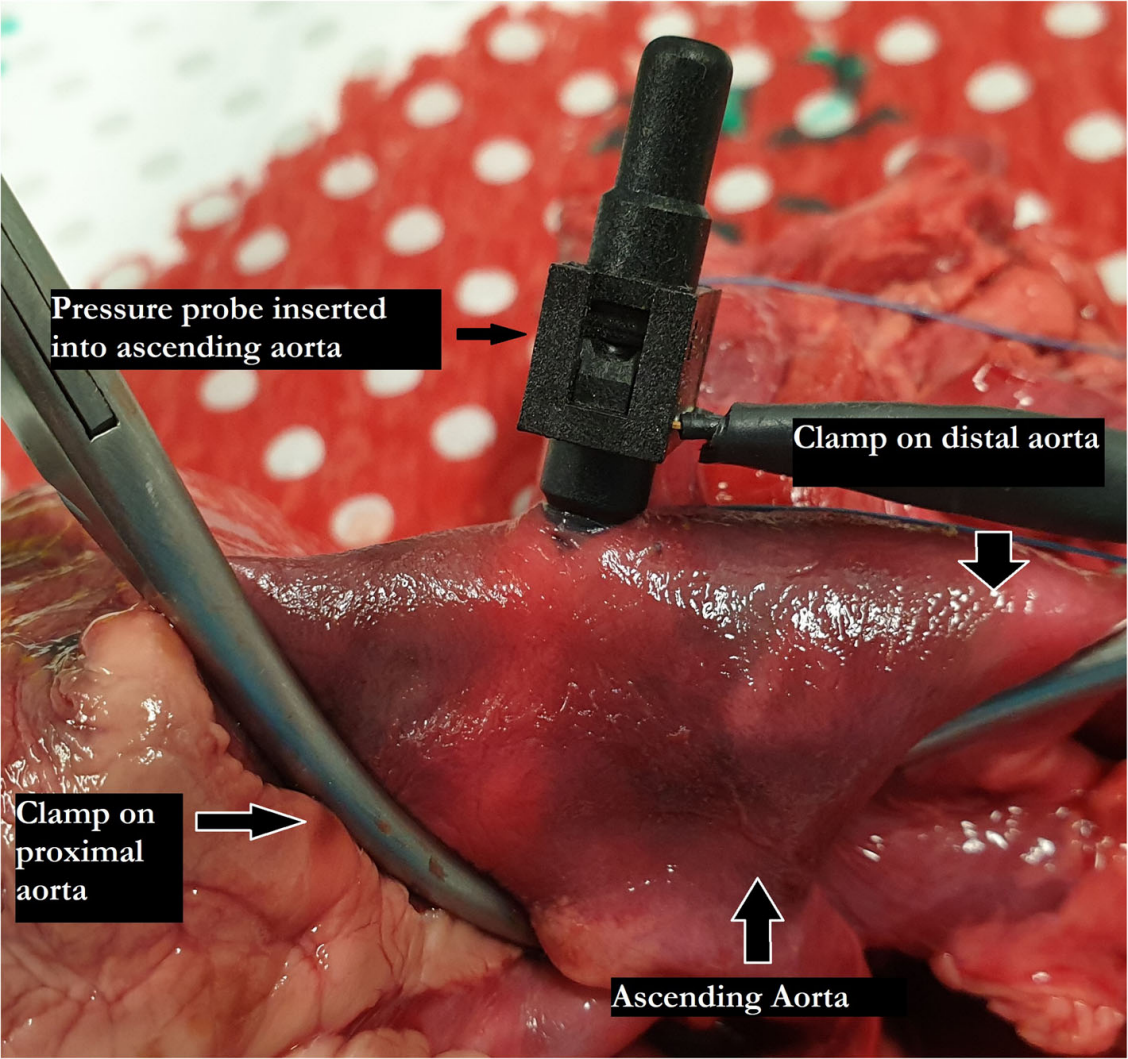
Aortic root and ascending aorta apparatus photograph. The proximal clamp is sitting at the most proximal portion of the aortic root clear of any aortic root structures. The pressure probe sits at the start of the proximal ascending aorta and distal clamp at the distal ascending aorta. Purse string sutures are yet to be placed around the pressure probe

**Fig. 3:**
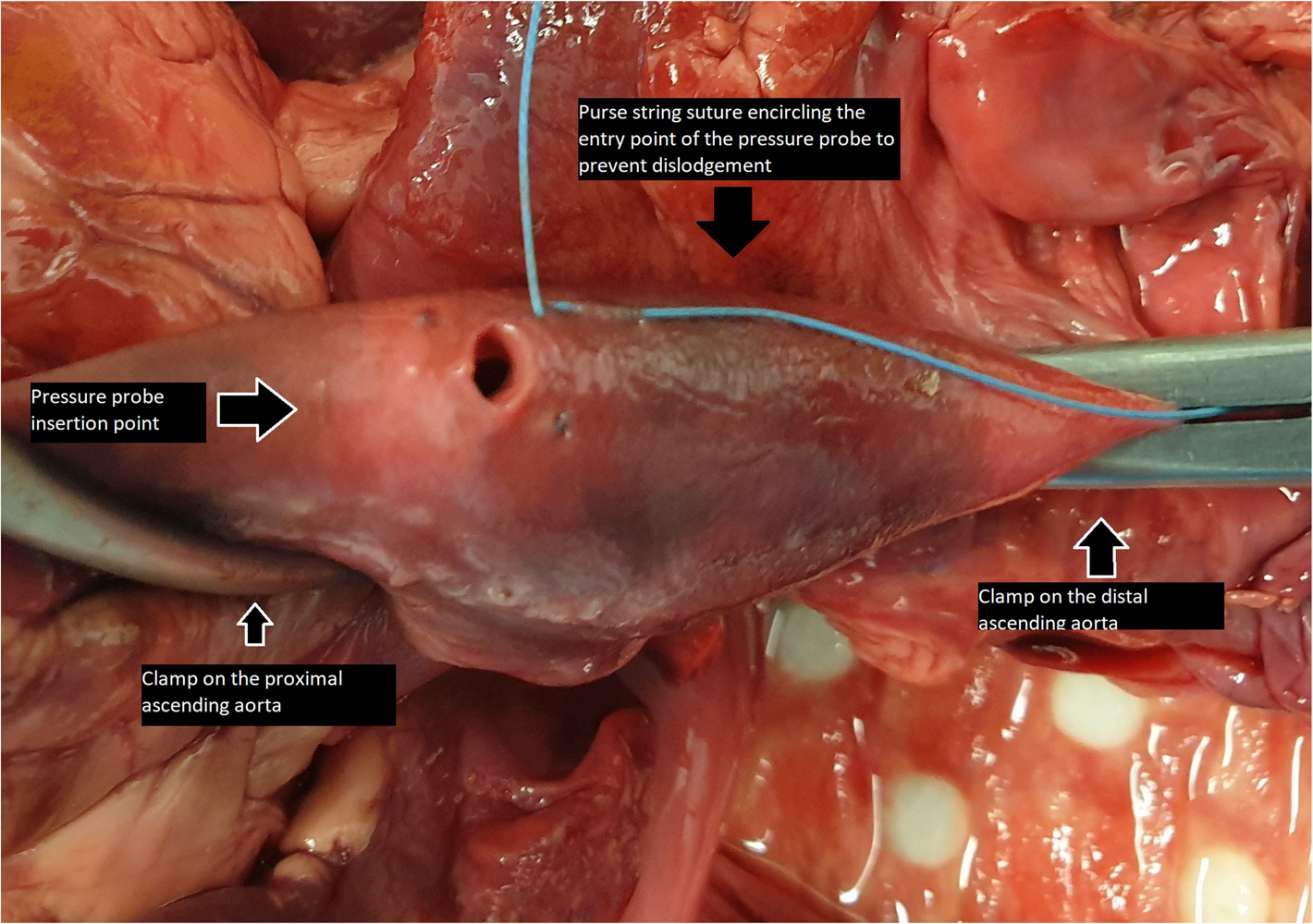
Overhead view of the aortic root and ascending aorta apparatus. The proximal clamp and distal clamp are at the proximal and distal limits of the thoracic aorta. The purse string suture is placed around the site of the pressure probe in the proximal ascending aorta

**Fig. 4:**
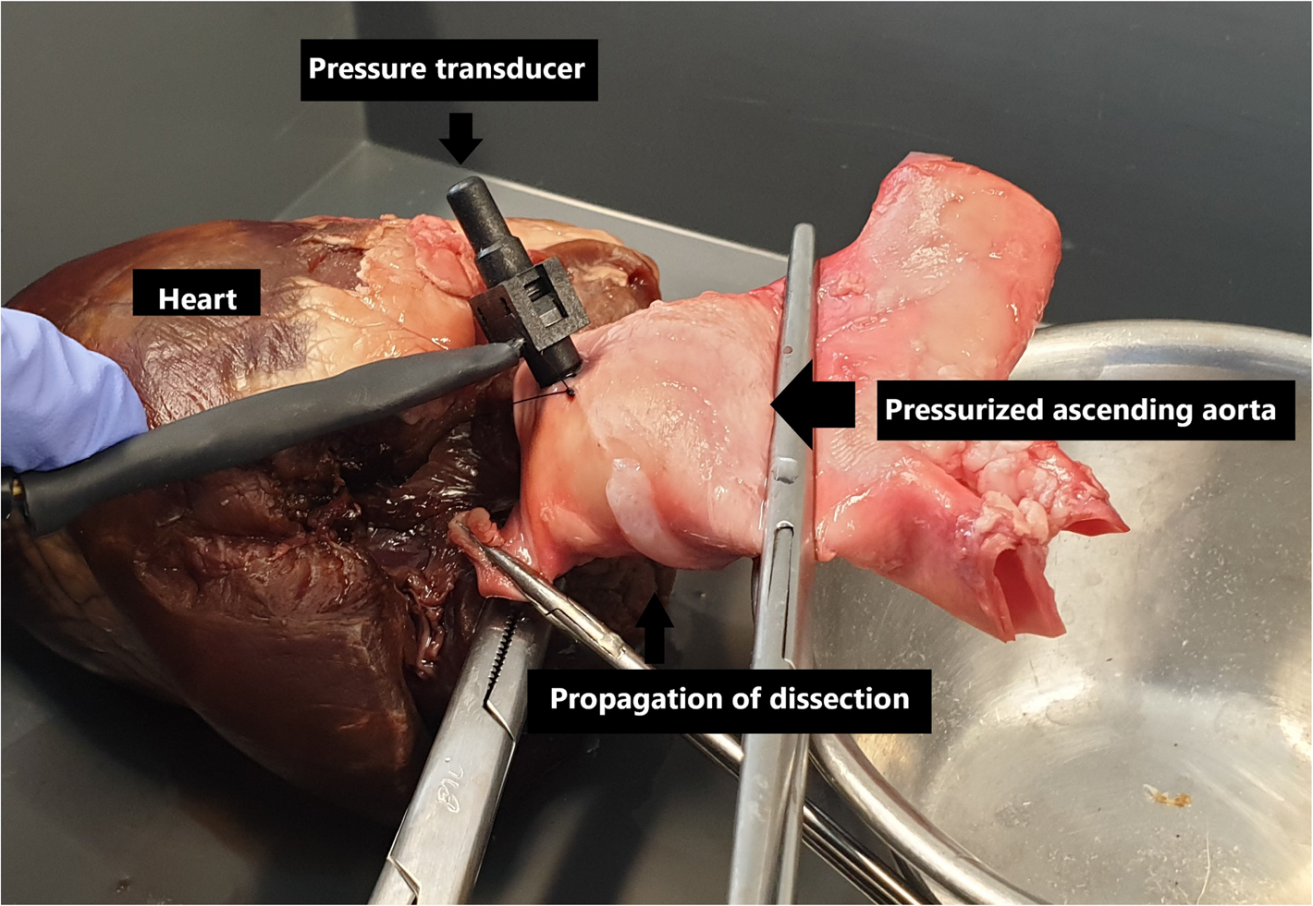
Photograph showing the aortic root and ascending aorta apparatus during pressure testing. The proximal clamp is positioned proximal to the aortic root, with small clamps placed on the left and right coronary arteries to prevent fluid leak. The pressure probe with associated purse string suture is positioned in the proximal ascending aorta, distal to the coronary arteries

Large vessel clamps were applied to the proximal and distal limits of the aorta (Figs. 2 and 3). The most distal region was limited by the branches of the aortic arch. The most proximal region limited by the left ventricle and careful avoidance of the aortic root structures and left and right coronary arteries. Using a size 11 scalpel blade, a small incision was made in the proximal ascending aorta distal to the aortic root, and the pressure transducer inserted within the ascending aorta lumen. A purse string suture was placed circumferentially around the incision to prevent dislodgement of the transducer during pressurization. The pressure transducer was connected to a laptop computer and pressure measurements taken in real time using LabVIEW (National Instruments Pty Ltd., Austin TX). Saline solution was aspirated into a 50 ml syringe and 21-gauge needle applied. The needle was then inserted between the intimal and medial layers at the level of the coronary ostia to create an aneurysm in the aortic root testing and in the region of the proximal aorta during the ascending aorta testing. Saline solution was administered until a visible aneurysm was created identifying disruption to the tissue layers. Using this same syringe and needle, saline solution was administered into the lumen of the ascending aorta between to distal and proximal clamps until the lumen was filled and pressurized. Concurrent pressure measurements (mmHg) were taken and recorded during filling (Fig. 4). Pressure measurements was taken at time of loss of tissue integrity, obvious tissue dissection or aneurysm rupture. The pressure measurement was determined to be the maximal pressure at time of loss of aortic root tissue integrity. The aortic root and ascending aorta was then opened, and the tissue microstructure was examined visually.

A limitation of this method of creating an aneurysm does not completely mirror the normal, chronic changes of aortic aneurysm formation including the thinning of the tissues, weakening of the connective tissues, and local stress points related to atherosclerosis (penetrating aortic ulcers) which could contribute to the development of aortic dissection.

## Results

**Fig. 5:**
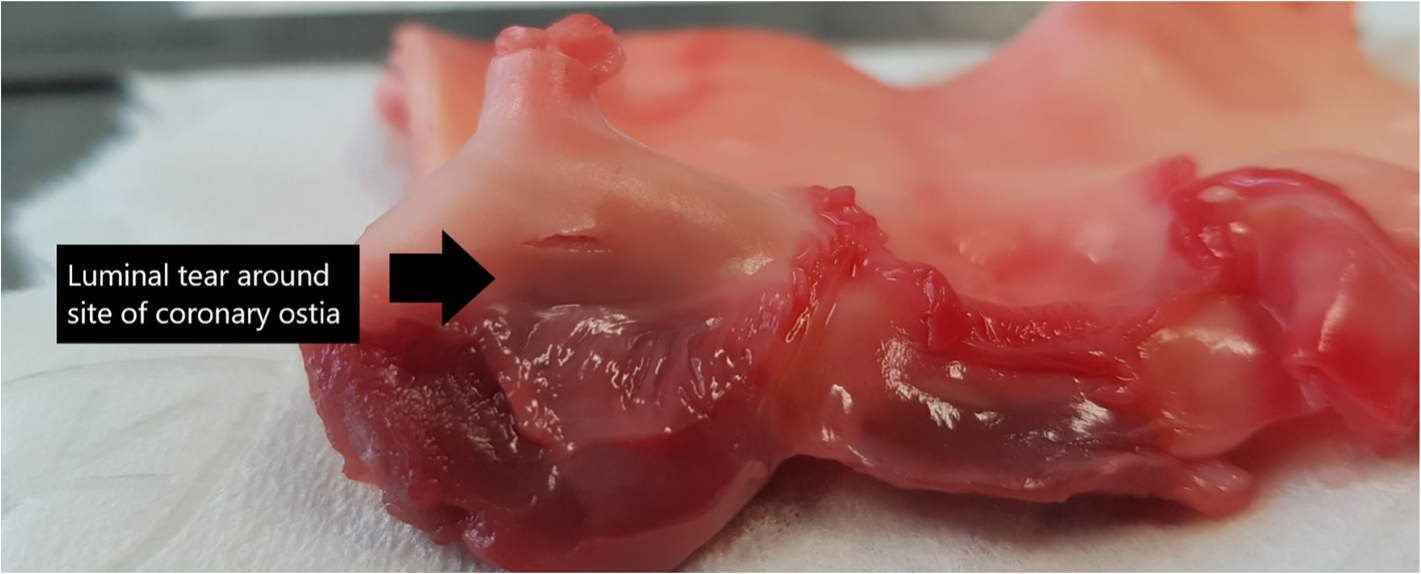
Photographs of the aortic root region cut open to examine the internal structures. What both photographs show are small tears in the lumen in the coronary ostia and sinus tissue regions as shown by the black arrow. The remaining valvular apparatus remained intact

**Fig. 6:**
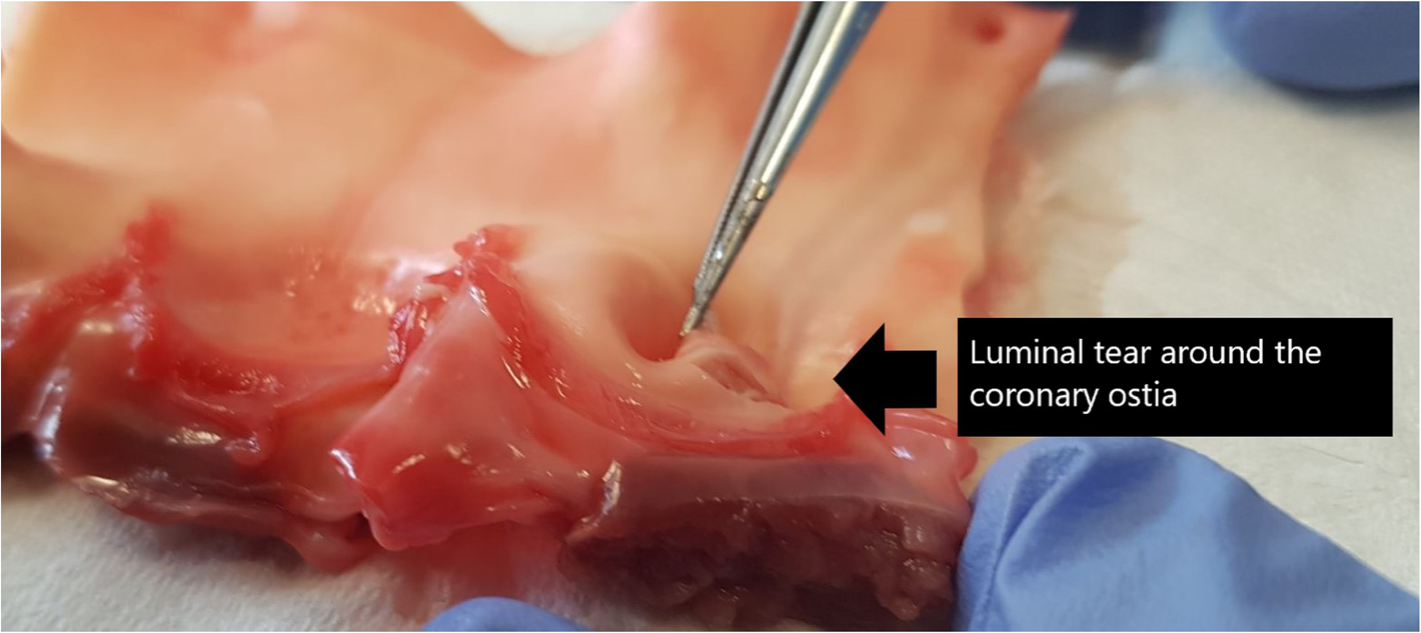
Photographs of the aortic root region cut open to examine the internal structures. What both photographs show are small tears in the lumen in the coronary ostia and sinus tissue regions as shown by the black arrow. The remaining valvular apparatus remained intact

Pressure measurements were conducted on 5 porcine aortic root specimens, and maximal pressure determined at the time of loss of tissue integrity. The mean maximal pressure (mmHg) at tissue failure was 208 mmHg (see Table 1). Macroscopic examination revealed luminal tears around the coronary ostia in 2/5 specimens (Figs. 5 and 6), and in all specimens, there was propagation of the dissection in the aortic root in a circumferential direction.

**Table 1:** Porcine pressure measurements of the aortic root and ascending aorta

Porcine specimen aortic root	Maximal pressure (mmHg)	Macroscopic characteristics	Porcine specimen ascending aorta only	Maximal pressure (mmHg)	Macroscopic characteristics
**1**	180	• Tissue dissection at site of pressure transducer • Circumferential spread of dissection	**1**	300+	• No loss of tissue integrity • Apparatus failure
**2**	200	• Tissue dissection at site of pressure transducer • Luminal tear at coronary ostia • Circumferential spread of dissection	**2**	300+	• No loss of tissue integrity • Apparatus failure
**3**	220	• Tissue dissection at site of pressure transducer • Luminal tear at coronary ostia • Circumferential spread of dissection	**3**	300+	• No loss of tissue integrity • Apparatus failure
**4**	200	• Tissue dissection at site of pressure transducer • Circumferential spread of dissection	**4**	300+	• No loss of tissue integrity • Apparatus failure
**5**	240	• Tissue dissection at site of pressure transducer • Circumferential spread of dissection	**5**	300+	• No loss of tissue integrity • Apparatus failure

**Fig. 7:**
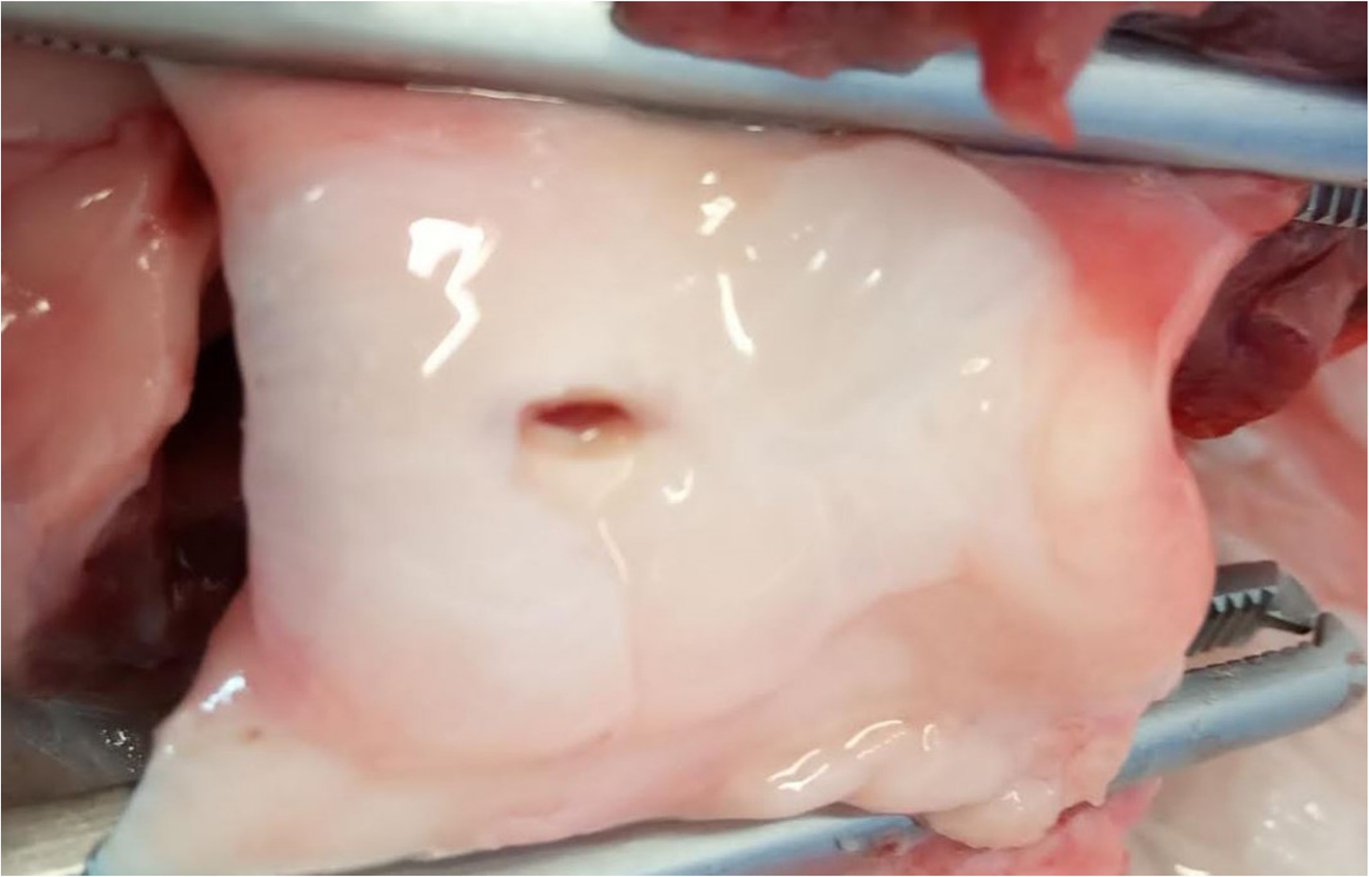
Photograph of the aortic root region taken from the superior aspect. The spreading of the injected saline into the aortic layers and propagating as a dissection in a circumferential pattern is seen. The superior clamp is proximal and the inferior clamp is distal. The pressure probe is removed from the center of the image for clarity

**Fig. 8:**
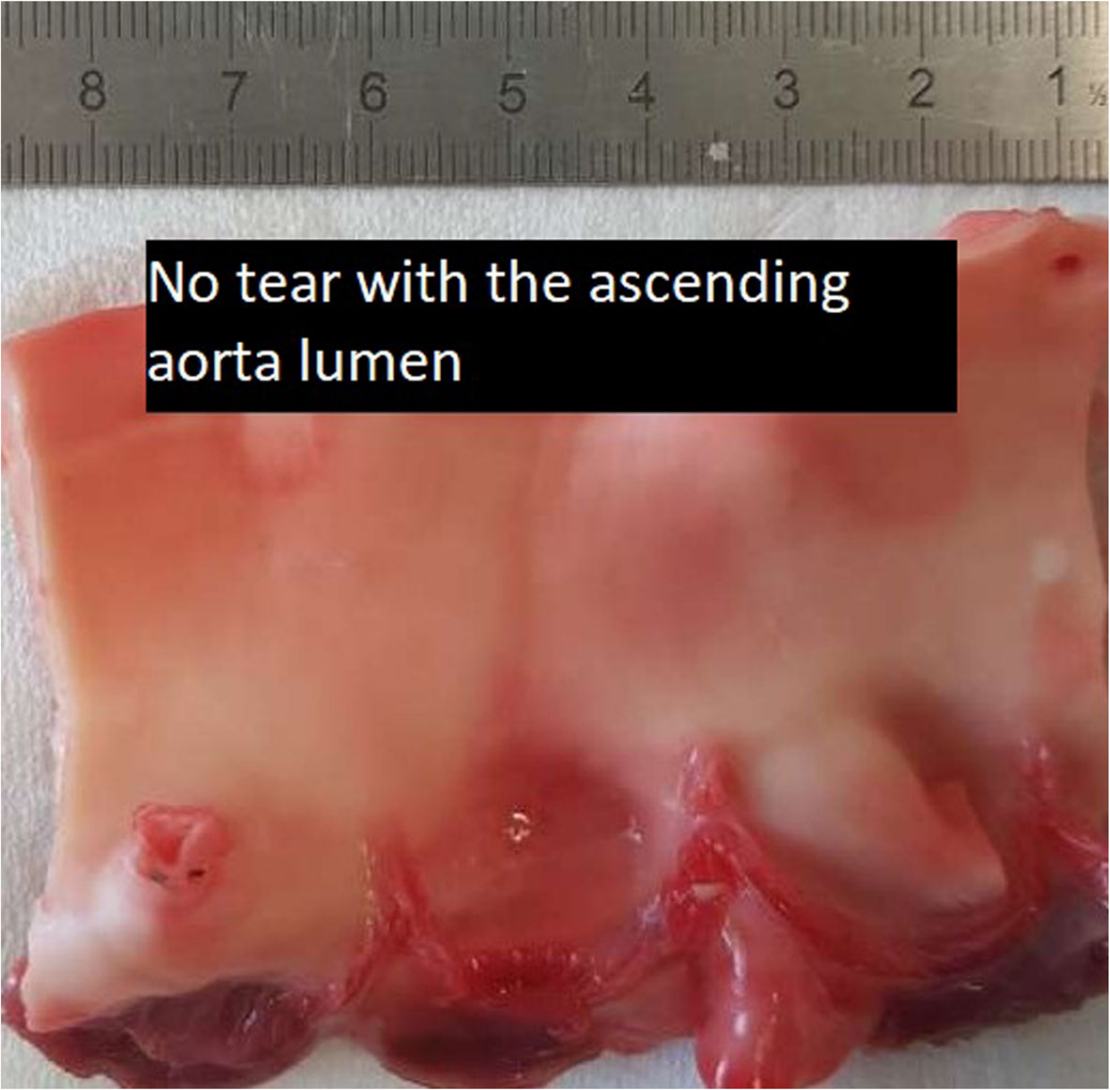
Photograph of the internal structures of the aortic root and ascending aorta following pressure testing. The photographs show an intact ascending aorta lumen with no tearing of the ascending aortic tissue in this test sample

Pressure measurements were conducted on 5 porcine ascending aorta specimens (excluding the aortic root), and maximal pressures recorded at the time of loss of tissue integrity or apparatus failure (Fig. 7). The median maximal pressure post rupture was 200 mmHg (range 180 to 240), compared to greater than 300 mmHg pre rupture for all specimens. This was significantly different. In all specimens, the maximal aortic pressures exceeded 300 mmHg without tissue failure or dissection, and eventual apparatus failure (see Table 1). Macroscopic examination revealed no luminal tissue dissection or tearing. There was no evidence of aneurysms dissection (Fig. 8).

## Discussion

The aortic root is a unique embryological, anatomical and physiological structure that should be distinguished from the ascending aorta in its diagnosis and surgical management of aortic root aneurysms. Diagnosis and subsequent management are determined by aneurysm size, progression of size and predisposing factors such as valvular pathology and genetic conditions such as Marfans syndrome and Loeys-Dietz syndrome. There are no reported studies comparing the macroscopic integrity of the aortic root in times of aneurysm pathology and its propensity to rupture at certain aortic pressures. All studies to date have looked at the aortic root and ascending aorta in section and not as a complete structure reducing its accuracy when compared to physiological conditions [9–15].

It has been reported that many dissection patients do not seem to have markedly dilated aortas at the time of presentation. On review of the International Registry of Aortic Dissections (IRAD) data, of 591 patients reviewed, almost 60% had diameters < 5.5 cm and 40% had aortic diameters < 5 cm. Suggestions have been made for utilization of genetic markers, biomarkers and functional studies to better predict susceptible patients to aortic dissection. If the aortic root represents a unique structure with a predisposition to rupture than the ascending aorta, then do we need even more aggressive monitoring, management and consideration for intervention in aneurysmal proximal ascending aorta and aortic root pathology?

We have looked at 10 porcine specimens comparing the aortic root and ascending aorta aneurysm rupture maximal pressures and rupture pattern. There are a number of limitations to our study. First, the use of a clamp at the most proximal part of the aortic root and a clamp in the most distal part of the ascending aorta may cause distortion to the aortic root and affect the pressures recorded. All attempts were made to place the proximal clamp devoid of any aortic root tissue in all experiments. Despite this limitation, clamping allowed for localization of the maximal pressure to a smaller area and precise administration of luminal fluid. Additionally, the ascending aorta pressure monitor required insertion into the proximal ascending aorta lumen itself causing disruption of the associated tissue structure. Although no major disruption of the tissue occurred at this site under high pressures, this may have been an area of weakness and minor fluid leak resulting in some skewing of obtained results.

Second, due to fresh porcine abattoir animal preparation prior to testing, significant mechanical injury was seen in the cardiac muscle and subsequently not amenable to use in the testing process. This required placement of the proximal clamp to prevent leaking of the intraluminal fluid through the cardiac internal and external tears.

Third, this static pressure model may not reflect the beating heart velocity of ventricular contraction (dp/dT) changes that occur in a clinical setting, but more reflects a measure of the pressure differences and tissue changes that occur under high luminal pressures in different parts of the thoracic aorta.

Simulation models have focused on a number of areas around the thoracic aorta including valvular function, aortic aneurysms, and aortic dissections [10, 16–24]. The studies listed have reported on the flow characteristics around the aortic valve particularly in patients with bicuspid valves and its effects on hemodynamics. Other simulations have focused on reproduction of the aortic aneurysm and dissection process using 3-dimensional (3D) aortic models derived from computer tomography (CT) scanning. Zannoli and colleagues in 2002, 2004, and most recently in 2007 [23] created a mechanical simulator to mock the cardiovascular system reproducing the frank-starling mechanism. Using a balloon and adjustable external reservoir with the aorta simulated by a rubber tubing, they aimed to create a device to reduce the high mortality in the presurgical phase of aortic dissections. They did this by three main mechanisms, improving coronary perfusion, slowing the dissection process, and recovering some of the mechanical efficiency of the cardiac-arterial junction [23]. The disadvantage of such approaches is the associated complexity and resources required to produce these models as well as the lack of gold standard validation in a number of cases.

Our results indicate that the aneurysmal aortic root tissues are at greater risk of rupture and dissection propagation at lower aortic pressure. Future testing of aortic root and ascending aorta pressure limits should include the incorporation of a dynamic pressure model using dp/dt and frank starling forces to replicate the cardiac cycle as accurately as possible. Further testing of greater tissue numbers is needed to confirm these findings, but consideration should be for much closer monitoring of aortic root aneurysms, strict blood pressure control of patients with known aortic root aneurysms and earlier intervention of aortic root aneurysms.

## Conclusion

The aortic root is a unique embryological, anatomical, and physiological structure that is shown to have specific development and progression of aneurysms, and as a result surgical management is different to that of the ascending aorta. No studies to date have tested the limitations of the weakened aortic root tissue, and we have reported on a reliable and reproducible aortic pressure model to identify the differences between these two structures. Knowledge in the pressure and structural limitations of the aneurysmal aortic root could guide clinical management of patients with known aneurysms, monitoring of progression and growth of aneurysms and ultimately surgical repair and replacement.

## Data Availability

All data incorporated into manuscript.

## Abbreviations

TAAD: Type A Aortic Dissection
ARR: Aortic root replacement
VSRR: Valve sparing root replacement
SAHMRI: South Australian Health and Medical Research Institute
PIRL: Preclinical, Imaging, and Research Laboratories
IRAD: International Registry of Aortic Dissections
Dp/dT: Derivative of pressure over time as a measure of cardiac contraction
CT: Computer tomography
3D: 3-dimensional
